# “Gilbert’s-like” syndrome as part of a spectrum of persistent unconjugated hyperbilirubinemia in post-chronic hepatitis patients

**DOI:** 10.1038/s41598-018-19847-4

**Published:** 2018-01-31

**Authors:** Jin Ye, Lianlian Cui, Yingqiao Zhou, Ying Huang, Omar Banafa, Xiaohua Hou, Zhen Ding, Rong Lin

**Affiliations:** Division of Gastroenterology, Union Hospital, Tongji Medical College, Huazhong University of Science and Technology, 430022 Wuhan, China

## Abstract

Gilbert’s syndrome (GS) patients present with remittent unconjugated hyperbilirubinemia. In this study, we investigated the correlation between polymorphisms in the gene encoding UDP-glucuronosyltransferase, *UGT1A1*, and the development of unconjugated hyperbilirubinemia in clinical GS and post-hepatitis hyperbilirubinemia. Blood samples were collected from 285 patients, including 85 patients who were clinically diagnosed with GS, 70 patients who had indirect hyperbilirubinemia during the recovery period of chronic liver diseases, 109 patients with normal hepatic function and 21 chronic active hepatitis patients. All samples were tested for the presence of the *28/*6 UGT1A1 genotype by pyrosequencing. Compared with the GS-control group, a significant difference in variations of the UGT1A1*28/*6 allele gene was found in GS patients. The post-hepatitis group showed a significant difference in the UGT1A1*28/*6 allele gene frequency distribution relative to that in the hepatitis control group. There were no significant differences between the GS group and post-hepatitis group in the distribution of the UGT1A1*28/*6 allele gene frequency and UGT1A1 diplotypes. UGT1A1*28/*6 gene polymorphisms in patients who had indirect hyperbilirubinemia while recovering from chronic liver diseases presented similar patterns as those seen for GS patients. These findings suggest that a “Gilbert’s-like” syndrome might be part of the spectrum of persistent unconjugated hyperbilirubinemia in post-chronic hepatitis patients.

## Introduction

Gilbert’s syndrome (GS) was first reported in 1901 by Augustin Gilbert and is characterized by remittent unconjugated hyperbilirubinemia due to partial or complete absence of bilirubin uridine diphosphate (UDP)-glucuronosyl-transferase 1 (UGT1) activity^1,2^. Bilirubin-UGT1 (UGT1A1) is the only UGT1 isoform that significantly contributes to the conjugation of bilirubin^3,4^. GS patients have hepatic UGT1A1 activity that is only approximately 30% that of normal hepatic tissues^5,6^.

UGT1 plays a key role in bilirubin metabolism, and UGT1A1*28/*6 gene polymorphisms carried by GS patients result in a UGT protein that has reduced activity^7^. The incidence of GS is approximately 15–25% in Africa, whereas lower rates (between 0–5% and 5–10%) are seen in Asian and Caucasian populations, respectively^8,9^. Meanwhile, UGT1A1 mutation types differ considerably among ethnic groups: a homozygous TA insertion in the TATA box (TA7) of the UGT1A1 promoter region (TA7/7) commonly occurs in Japanese and Caucasian GS patients^10^, whereas UGT1A1*6(G71R), but not UGT1A1(TA7/7), is commonly seen in Taiwanese, Korean and Japanese patients^11,12^. The significance of the different UGT1A1 mutations and specificity of gene polymorphisms among GS patients of different ethnic groups is unclear. It revealed the ethnic specificity of the UGT1 genetic diversity in healthy Chinese populations compared with those of populations in Japan, as well as African and European countries^13^, but there is limited information concerning the general genetic variation of UGT1A1 in GS patients in Chinese populations. Moreover, the influence of these polymorphisms and whether GS involves single or multiple factors are unclear.

After receiving effective treatments for acute and chronic liver disease, most patients present with a decline in liver enzyme indices, and bilirubin levels also gradually return to normal during the recovery period. However, persistent or intermittent mild unconjugated hyperbilirubinemia can be detected in many patients with chronic persistent hepatitis. For these patients, there is no indication for the treatment for liver inflammation because the liver enzyme indices are usually normal, viral DNA is undetectable, and ultrasound tests often do not indicate liver disease. Routine treatments for jaundice are usually not effective for these patients, and persistent unconjugated hyperbilirubinemia may seriously affect the quality of life for some patients. Such patients are often classified as having so-called “post-hepatitis hyperbilirubinemia”. Felsher *et al*. demonstrated that the mean UGT1A1 activity was significantly lower in 12 patients with chronic persistent hepatitis, and these patients also presented with persistent or intermittent mild unconjugated hyperbilirubinemia compared with healthy individuals^14^. However, the number of cases in this study was small; thus, the possible involvement of UGT1A1 in post-hepatitis hyperbilirubinemia remains unclear.

GS and chronic persistent liver disease are two distinct diseases. UGT1A1 activity has been linked to the development of indirect hyperbilirubinemia in both clinical GS and post-hepatitis hyperbilirubinemia, raising the question of whether “Gilbert’s-like” aberrations in bilirubin metabolism are part of the spectrum of hyperbilirubinemia in post-chronic persistent hepatitis and what is the role of gene UGT1A1 polymorphisms in this condition. In this study, we investigated the correlation between UGT1A1 gene polymorphisms and the development of unconjugated hyperbilirubinemia in GS and post-hepatitis hyperbilirubinemia, and provide a new strategy for the possible aetiology, pathogenesis and therapy for “Gilbert’s like” syndrome in persistent or intermittent mild unconjugated hyperbilirubinemia in post-chronic liver disease.

## Results

### Association of the bilirubin level and UGT1A1 polymorphisms

**Figure 1:**
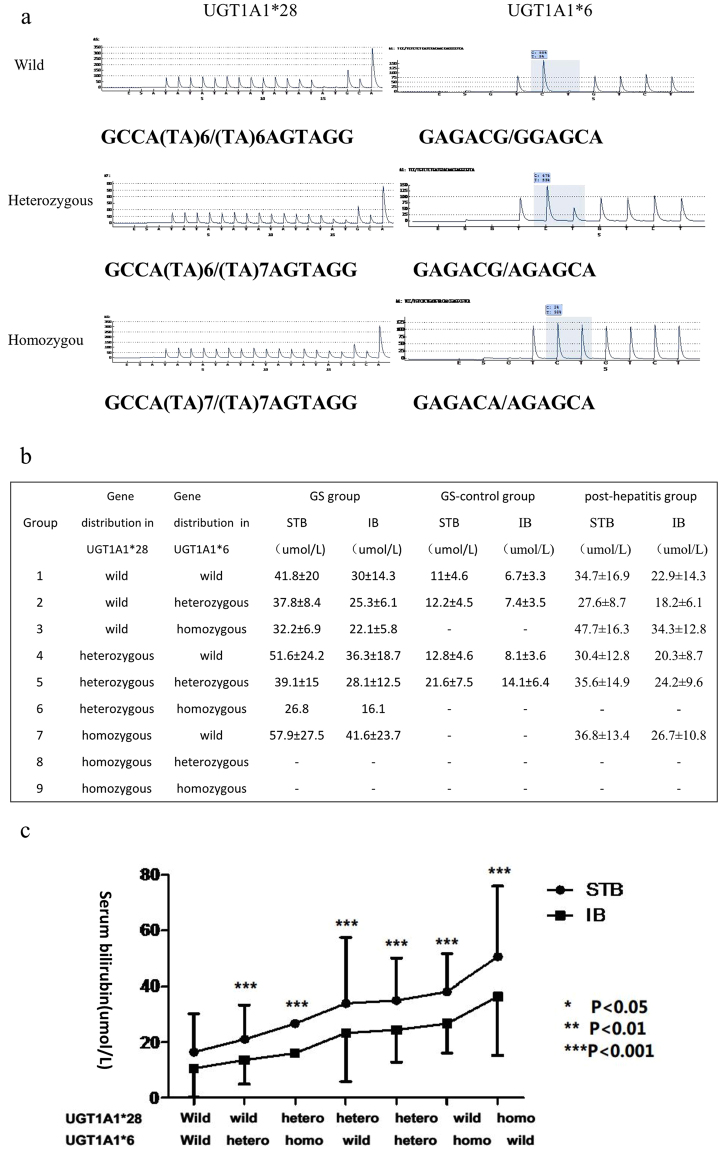
STB/IB levels correlate with the presence of UGT1A1*28/*6 polymorphisms. (**a**) Sequences of UGT1A1*28/*6 polymorphisms; (**b**) STB/IB levels for different UGT1A1 genotypes in the GS, GS-control and post-hepatitis groups; (**c**) STB/IB levels for different UGT1A1 genotypes indicated significant differences (P = 0.000). The STB level was markedly elevated when individuals were homozygous for UGTA1*28 or *6. STB: serum total bilirubin; IB: indirect bilirubin.

This study examined 285 individuals who were divided into four groups: (i) 85 Gilbert’s Syndrome (GS) patients; (ii) healthy controls (n = 109); (iii) post-hepatitis hyperbilirubinemia patients (n = 70); and (iv) hepatitis control (n = 21). UGT1A1*28 and UGT1A1*6 polymorphisms were found in Chinese GS subjects (See Fig. 1a and Supplementary Table S1 for sequences and genotypes, respectively). Subjects with UGT1A1*28 and UGT1A1*6 polymorphisms showed a wide range in the total serum bilirubin (STB) level. The STB level was increased in the following order: wild UGT1A1*28 combined with wild UGT1A1*6 (UGT1A1*28 wild/UGT1A1*6 wild) < UGT1A1*28 wild/UGT1A1*6 hetero < UGT1A1*28 hetero/UGT1A1*6 homo < UGT1A1*28 hetero/UGT1A1*6 wild < UGT1A1*28 hetero/UGT1A1*6 hetero < UGT1A1*28 wild/UGT1A1*6 homo < UGT1A1*28 homo/UGT1A1*6 wild (Fig. 1b,c). Overall, the STB level was markedly elevated when individuals were homozygous for UGTA1*28 or *6.

### UGT1A1 polymorphisms and GS

A homozygous TA insertion in the TATA box of the UGT1A1 promoter region (UGT1A1*28) (22%) and homozygous UGT1A1*6 (14%) were frequently seen in GS patients. Meanwhile, no homozygous genotype for either UGT1A1*6 and UGT1A1*28 was seen in the control patients. The gene allele frequency of UGT1A1 was significantly elevated in GS patients compared with that in the control patients (UGT1A1*28: 0.465 vs 0.101; UGT1A1*6: 0.288 vs 0.142; p < 0.05, Table 1). All the GS patients (n = 85) were grouped into 7 different UGT1A1*28 combined with UGT1A1*6 genotypes (Table 2). There were 4 subtypes in the control patient group. The frequency of UGT1A1*28 wild/UGT1A1*6 wild was 5.9% in the GS and 56% in the GS control group (odds ratio(OR): 0.05; Chi-square: 53.36, p < 0.001, Table 2). The frequency of UGT1A1*28 wild/UGT1A1*6 hetero, UGT1A1*28 hetero/UGT1A1*6 hetero and UGT1A1*28 hetero/UGT1A1*6 wild also differed between the GS and control groups. UGT1A1*28 wild/UGT1A1*6 homo, UGT1A1*28 hetero/UGT1A1*6 homo and UGT1A1*28 homo/UGT1A1*6 wild, were not detected in the control patients (Table 2). The distribution of UGT1A1*28 and UGT1A1*6 polymorphisms was significantly different between the GS and GS-control groups.

**Table 1:** Characteristics of UGT1A1*28/*6 polymorphisms in all groups.

Allele gene frequency	GS group	GS-control group	post-hepatitis group	hepatitis-control group
UGT1A1*28	0.465*	0.101*	0.35**	0.143**
UGT1A1*6	0.288^#^	0.142^#^	0.286^##^	0.119^##^

The gene allele frequency of UGT1A1 was significantly elevated in GS patients compared with that in the control patients (UGT1A1*28: 0.465 vs 0.101, *p = 0.000; UGT1A1*6: 0.288 vs 0.142; #p = 0.000).

The gene allele frequency of UGT1A1 was significantly elevated in post-hepatitis hyperbilirubinemia patients compared with that in hepatitis-control patients (UGT1A1*28: 0.350 vs 0.143, **p = 0.031; UGT1A1*6: 0.286 vs 0.119; ##p = 0.049).

**Table 2:** Characteristics of the correlation between UGT1A1*28/*6 polymorphisms and GS.

Group	Total cases	UGT1A1*28(w)	UGT1A1*28(w)	UGT1A1*28(hetero)	UGT1A1*28(hetero)	UGT1A1*28(w)	UGT1A1*28(homo)	UGT1A1*28(hetero)
		UGT1A1*6(w)	UGT1A1*6(hetero)	UGT1A1*6(w)	UGT1A1*6(hetero)	UGT1A1*6(homo)	UGT1A1*6(w)	UGT1A1*6(homo)
GS	85	5 (5.9%)	9(10.6%)	24(28.2%)	16(18.8%)	11(12.9%)	19(22.4%)	1(1.2%)
GS-Con	109	61 (56%)	26(23.8%)	17(15.6%)	5(4.6%)	0	0	0
OR value		0.05	0.38	2.13	4.82	—	—	—
95% CI		0.18~0.13	0.17~0.86	1.06~4.29	1.69~13.77	—	—	—
Chi-square		53.36	5.68	4.58	10.03	—	—	—
P value		0	0.02	0.03	0	—	—	—

The distribution of the UGT1A1*28/*6 genotypes was significantly different between the GS and GS-control group (P < 0.05).

### UGT1A1 polymorphisms and post-hepatitis hyperbilirubinemia

Homozygous TA insertion in the TATA box of the promoter region (UGT1A1*28) (14%) and homozygous UGT1A1*6 (11%) were frequently seen in the post-hepatitis patient group. Among the hepatitis-control patients, none were homozygous for the UGT1A1*6 genotype, and only one was homozygous for the UGT1A1*28 genotype. The gene allele frequency of UGT1A1 was significantly elevated in post-hepatitis hyperbilirubinemia patients compared with that in hepatitis-control patients (UGT1A1*28: 0.350 vs 0.143; UGT1A1*6: 0.286 vs 0.119; p < 0.05, Table 1). Six different sub-genotypes based on UGT1A1*28 combined with UGT1A1*6 were detected in post-hepatitis patients (n = 70), and there were 5 subtypes seen among the hepatitis control group patients. The frequency of UGT1A1*28 wild/UGT1A1*6 wild was 15.8% in the post-hepatitis group and 57.1% in the hepatitis control group (OR: 0.14; Chi-square: 14.68, p < 0.001, Table 3).

**Table 3:** Characteristics of the UGT1A1 genotype distribution in the post-hepatitis hyperbilirubinemia group and hepatitis-control group.

Group	Total cases	UGT1A1*28(w)	UGT1A1*28(w)	UGT1A1*28(hetero)	UGT1A1*28(hetero)	UGT1A1*28(homo)	UGT1A1*28(w)
		UGT1A1*6(w)	UGT1A1*6(hetero)	UGT1A1*6(w)	UGT1A1*6(hetero)	UGT1A1*6(w)	UGT1A1*6(homo)
Post-hepatitis	70	11 (15.8%)	12 (17.1%)	17 (24.3%)	12 (17.1%)	10 (14.3%)	8 (11.4%)
Hepatitis-Con	21	12 (57.1%)	4 (19%)	3 (14.3%)	1 (4.8%)	1 (4.8%)	0
OR value		0.14	0.88	1.93	4.14	6	—
95% CI		0.05~0.41	0.25~3.08	0.51~7.34	0.51~33.87	0.73~49.2	—
Chi-square		14.68	0.04	—	—	—	—
P value		0	0.84	—	—	—	—

The distribution of the UGT1A1*28/*6 genotypes differed significantly between the post-hepatitis hyperbilirubinemia group and hepatitis-control group.

### Genotype characteristics in the GS and post-hepatitis hyperbilirubinemia groups

All the GS patients (n = 85) could be grouped into 7 different UGT1A1*28 combined with UGT1A1*6 genotypes. Among the control group individuals, there were 4 genotype subtypes. The frequency of UGT1A1*28 homo/UGT1A1*6 wild was 22.4% in the GS and 14.3% in the post-hepatitis group (OR: 1.73; Chi-square: 1.64, p > 0.05, Table 4). The frequency of UGT1A1*28 wild/UGT1A1*6 homo was 12.9% in the GS group and 11.4% in the post-hepatitis group (OR: 1.15; Chi-square: 0.08, p > 0.05, Table 4). The frequency of UGT1A1*28 wild/UGT1A1*6 wild, UGT1A1*28 wild/UGT1A1*6 hetero, UGT1A1*28 hetero/UGT1A1*6 hetero and UGT1A1*28 hetero/UGT1A1*6 wild also presented with a similar pattern between post-hepatitis patients and GS patients (Table 4).

**Table 4:** Characteristics of the UGT1A1*28/*6 genotypes in the GS and post-hepatitis hyperbilirubinemia groups.

Group	Total cases	UGT1A1*28(w)	UGT1A1*28(w)	UGT1A1*28(hetero)	UGT1A1*28(hetero)	UGT1A1*28(homo)	UGT1A1*28(w)	UGT1A1*28(hetero)
		UGT1A1*6(w)	UGT1A1*6(hetero)	UGT1A1*6(w)	UGT1A1*6(hetero)	UGT1A1*6(w)	UGT1A1*6(homo)	UGT1A1*6(homo)
GS	85	5 (5.9%)	9(10.6%)	24(28.2%)	16(18.8%)	19(22.4%)	11(12.9%)	1(1.2%)
Post-hepatitis	70	11 (15.8%)	12 (17.1%)	17 (24.3%)	12 (17.1%)	10 (14.3%)	8 (11.4%)	0
OR value		0.45	0.57	1.23	1.12	1.73	1.15	—
95% CI		0.15~1.36	0.23~1.45	0.60~2.53	0.49~2.56	0.74~4.01	0.44~3.04	—
Chi-square		2.1	1.41	0.31	0.07	1.64	0.08	—
P value		0.15	0.24	0.58	0.79	0.2	0.78	—

UGT1A1*28/*6 polymorphisms present similar patterns in post-hepatitis patients and GS patients (P > 0.05), whereas chronic active hepatitis patients showed a different pattern.

## Discussion

Our study investigated the characteristics of *UGT1A1* polymorphisms in Chinese patients with post-hepatitis hyperbilirubinemia and Gilbert’s syndrome (GS). UGT1A1*28/*6 gene polymorphisms are correlated with the development of unconjugated hyperbilirubinemia in both clinical GS and post-hepatitis hyperbilirubinemia. Patients carrying the UGT1A1*28/*6 gene polymorphism who had indirect hyperbilirubinemia while recovering from chronic liver diseases can present with a pattern similar to that seen for GS patients.

Although UGT1 genetic diversity in the healthy Chinese population has been reported and GS is correlated with UGT1A1*28/*6 gene polymorphisms^7,15–18^, there is limited information concerning the genetic variation of UGT1A1 in Chinese GS patients. The influence of genetic variations and other factors, whether alone or combined, on GS pathology is unclear. Our study found that the gene allele frequency of UGT1A1 *28 was 0.465 and 0.101 in the GS group and control group, respectively (Table 1). Meanwhile, the allele gene frequency of the UGT1A1 *6 mutation was 0.288 and 0.142 in the GS group and control group, respectively (Table 1). Compared with the GS-control group, a significant difference in the variation of the UGT1A1*28/*6 allele gene was found in GS patients (P < 0.05).

During the recovery period, the liver enzyme indices and serum bilirubin levels of hepatitis patients were typically reduced gradually to normal levels. However, similar to GS patients, persistent or intermittent mild unconjugated hyperbilirubinemia can be detected in post-hepatitis patients. The possible pathogenesis of post-hepatitis hyperbilirubinemia remains unclear, and there is limited clinical guidance for clear diagnostic criteria and therapy for these patients, which can result in patient and physician confusion as well as variations in treatment practices. Our results indicated a significant difference in the UGT1A1*28/*6 allele gene frequency distribution in the post-hepatitis hyperbilirubinemia group compared with that in the hepatitis-control group, but there was no significant difference in UGT1A1*28/*6 gene polymorphisms between the GS and post-hepatitis hyperbilirubinemia groups. The gene polymorphisms of UGT1A1*28/*6 in patients who had indirect hyperbilirubinemia during the recovery period from chronic liver diseases presented with a similar pattern to that of GS patients but differed from that for chronic active hepatitis patients.

Although GS patients may experience hyperbilirubinemia, treatment generally is not necessary^19,20^. Pyrosequencing of UGT1A1*28/*6 gene polymorphisms could thus be used as an effective auxiliary method for GS diagnosis after excluding other possible causes of indirect hyperbilirubinemia. Persistent or intermittent unconjugated hyperbilirubinemia could also present in patients during recovery from chronic liver diseases. If these patients carry a UGT1A1 mutation, we would recommend that they not be treated. The pyrosequencing assay for UGT1A1*28/*6 gene polymorphisms has a certain degree of specificity and sensitivity^21^ and may provide evidence for the clinical diagnosis and treatment of GS and post-hepatitis hyperbilirubinemia.

In conclusion, UGT1A1*28/*6 gene polymorphisms are correlated with the development of unconjugated hyperbilirubinemia in clinical GS and post-hepatitis hyperbilirubinemia. Clinicians should be aware that “Gilbert’s-like” syndrome might be part of a spectrum characterizing persistent unconjugated hyperbilirubinemia in post-chronic hepatitis patients.

## Materials and Methods

### Subjects

Blood samples were collected from 285 hospitalized patients and outpatients who were treated between August 2012 and February 2015 at Wuhan Union Hospital, Tongji Medical College, Huazhong University of Science and Technology. All the study participants were members of the Han population. Eighty-five patients were clinically diagnosed as having Gilbert’s Syndrome (GS; male: n = 67, female: n = 18, average age 35 ± 14 yrs.). The diagnostic criteria for GS were as follows: (1) a history of intermittent jaundice lasting more than six months that worsened with fatigue, alcohol consumption, infection, menstruation or stress; (2) an unconjugated bilirubin and conjugated bilirubin level that was normal or less than 20 percent of total bilirubin, respectively; a serum AST level that was normal, with the exception of other liver diseases and haemolysis; (3) the absence of other diseases that could cause elevated unconjugated bilirubin levels such as hyperthyroidism or drug side effects; (4) the absence of Crigler-Najjar syndrome types I and II. In addition, 109 patients with normal hepatic function served as the GS control group (male: n = 58, female: n = 51, average age 53 ± 12 yrs.). All the control individuals denied a history of jaundice, abnormal liver function and liver disease. The post-hepatitis hyperbilirubinemia group included 70 patients (male: n = 52, female: n = 18, average age: 41 ± 13 yrs.) who had unconjugated hyperbilirubinemia while recovering from chronic hepatic diseases (chronic hepatitis b, viral hepatitis: n = 30, patients with hepatitis b cirrhosis: n = 16, 3 patients with alcoholic liver cirrhosis: n = 3, chronic alcoholic liver disease: n = 6, fatty liver: n = 13, chronic schistosomiasis liver disease: n = 2). The hepatitis-control group included 21 chronic active hepatitis patients (male: n = 19, female: n = 2, average age: 48 ± 12 yrs.). Clinical information was recorded in detail for all four patient groups. The study was approved by the Ethics Committee of Tongji Medical College, Huazhong University of Science and Technology. The study methods were carried out in accordance with approved guidelines, and all the subjects provided written informed consent prior to study enrolment.

### Extraction of genomic DNA and gene sequencing

Peripheral venous blood samples (3 mL) were transferred to EDTA anticoagulant tubes with sufficient anticoagulation. A Wizard Genomic DNA Purification Kit was used to extract and purify genomic DNA according to the manufacturer’s protocol (Promega, Madison, WI, USA). DNA purity was confirmed with an A_260_/A_280_ ratio >1.7. DNA samples were stored at 2–8 °C until use. For UGT1A1 *28 (A(TA)7TAA (UGT1A1*28/*28; 7/7)), the upstream primer sequence was 5′- TCCCTGCTACCTTTGTGGAC-3′, The downstream primer sequence was 5-GAGGTTCGCCCTCTCCTACT-3′; UGT1A1*6(A(TA)6TAA (UGT1A1*1/*1;6/6)) the upstream primer sequence was 5′-GAGGTTCTGGAAGTACTTTG-3′ and the downstream primer sequence was 5′-CCAGAGGTTCGCCCTCTCCTACT-3′. The primers were provided by Changshasanji Biological TechnologyCo.,Ltd, (Changsha,China). The PCR mixture included 2 μl of genomic DNA solution, 1 μl of each primer, 0.5 U of Taq DNA polymerase TaKaRa corporation (Dalian), 3 μl of 4× dNTP, and 5 μl of 10× buffer (500 mmol/L KCl; 100 mmol/L Tris-HCl (pH 8.3); 15 mmol/L MgCl_2_, 0.1% gelatin), in a total volume of 50 μl. Amplification was performed using the following cycling conditions: 95 °C for 10 min, 35 cycles of 95 °C for 30 s, specific annealing at 60 °C for 30 s and 72 °C for 30 s, and a final extension for 7 min at 72 °C. The pyrosequencing assay for the UGT1A1 *28 and UGT1A1 *6 gene polymorphisms was performed using the PyroMark Q24 system according to the manufacturer’s instructions (Qiagen).

### Statistical analysis

The data are presented as the means ± standard error of the mean (SEM). Statistical analysis was performed using SPSS statistical software version 19.0 using either Chi-square, contingency table or Fisher’s exact probability method depending on the dataset. A p < 0.05 indicated statistical significance.

## Electronic supplementary material

Supplementary information
