# Supplementary material for: “Gilbert’s-like” syndrome as part of a spectrum of persistent unconjugated hyperbilirubinemia in post-chronic hepatitis patients
Source: Sci Rep. 2018 Jan 31;8:2008. doi: 10.1038/s41598-018-19847-4 (PMC5792633; doi:10.1038/s41598-018-19847-4)
Supplement: Supplementary file 1 — Supplementary information [file 41598_2018_19847_MOESM1_ESM.pdf]

"Gilbert's-like" syndrome as part of a spectrum of persistent unconjugated hyperbilirubinemia in post-chronic hepatitis patients

Jin Ye, Lianlian Cui, Yingqiao Zhou,Ying Huang, Omar Banafa, XiaohuaHou, Zhen Ding, Rong Lin *

Supplementary Table S1 The genotypes and gene polymorphisms of UGT1A1*28/*6

	Gene polymorphisms	Genotype
	$(TA)_6/(TA)_6$	wild
UGT1A1 *28（［TA］n）	$(TA)_6/(TA)_7$	heterozygous
	$(TA)_7/(TA)_7$	homozygous
	GG	wild
UGT1A1*6（211 G>A）	GA	heterozygous
	AA	homozygous
